# Glucocorticoid Insensitivity in Severe Asthma: Underlying Molecular Mechanisms, Challenges, and Emerging Therapies

**DOI:** 10.26502/aimr.0202

**Published:** 2025-04-11

**Authors:** Chang Kon Kim, Devendra K. Agrawal

**Affiliations:** 1 Department of Translational Research, College of Osteopathic Medicine of the Pacific, Western University of Health Sciences, Pomona, California 91766 USA.

**Keywords:** Airway Hyperresponsiveness, Airway Remodeling, Biologic Therapies, Glucocorticoid Insensitivity, Glucocorticoid Receptor Signaling, Inflammation, Inhaled corticosteroids, Interleukins, Janus Kinase Inhibitors, NF-κB Activation, Severe Asthma

## Abstract

Glucocorticoids are the cornerstone of asthma therapy due to their potent anti-inflammatory action. However, a subset of severe asthmatics do not respond to the standard glucocorticoid treatment. Such phenomenon is referred to as glucocorticoid insensitivity (GCI). From a clinical point of view, GCI is characterized by the reduced therapeutic response with improvement of less than 10–15% in lung function parameters, such as FEV1, upon the administration of an adequate glucocorticoid dose. The mechanisms underlying GCI involve disrupted glucocorticoid receptor (GR) signaling, overexpression of the dominant-negative GRβ isoform, increased activity of pro-inflammatory transcription factors such as NF-κB and AP-1, and abnormal GR phosphorylation by kinases such as p38 MAPK. These altered molecular pathways undermine the anti-inflammatory effects of glucocorticoids on immune and structural airway cells, thus maintaining the chronicity of airway inflammation and remodeling. GCI can be of innate genetic origin, as in the case of GR mutations, or acquired through environmental exposures, including viral infections, smoking, and long-term exposure to pollutants in the environment. GCI represents a big challenge in the management of asthma, since a large proportion of cases do not achieve an adequate level of control with the standard treatment options. Recent advances in the understanding of the molecular mechanisms underlying GCI have enabled the development of novel therapeutic strategies, including biologic therapies targeting interleukin-5 and IL-13, Janus kinase inhibitors, and small-molecule drugs aimed at restoring GR function. This article presents a critical discussion on the current state of knowledge regarding the glucocorticoid resistance mechanisms in asthma, identifying the clinical effects of new therapeutic strategies, with special emphasis on the need for personalized treatment regimens to improve outcomes in glucocorticoid insensitivity.

## Introduction

Bronchial asthma is a chronic inflammatory disease of the airways that affects over 300 million people worldwide [1]. The prevalence of this disease has been steadily rising, particularly in the urban areas of developing countries. Wheezing, dyspnea, chest tightness, and coughing result from airway hyperresponsiveness, inflammation, and obstruction; these symptoms vary from one patient to another [2–4]. Symptoms are generally induced by various stimuli, such as allergens, infections, and environmental pollutants, which underlines the heterogeneity of asthma. Severe asthma represents 5–10% of all asthma and poses major challenges because of the resistance to standard therapies, including glucocorticoids (GCs). Patients with severe asthma frequently develop numerous exacerbations and sustained impairment in their quality of life, pointing to the necessity of developing new options for treatment [5].

### Pathophysiology and Underlying Cellular and Molecular Mechanism of Asthma

**Figure 1:**
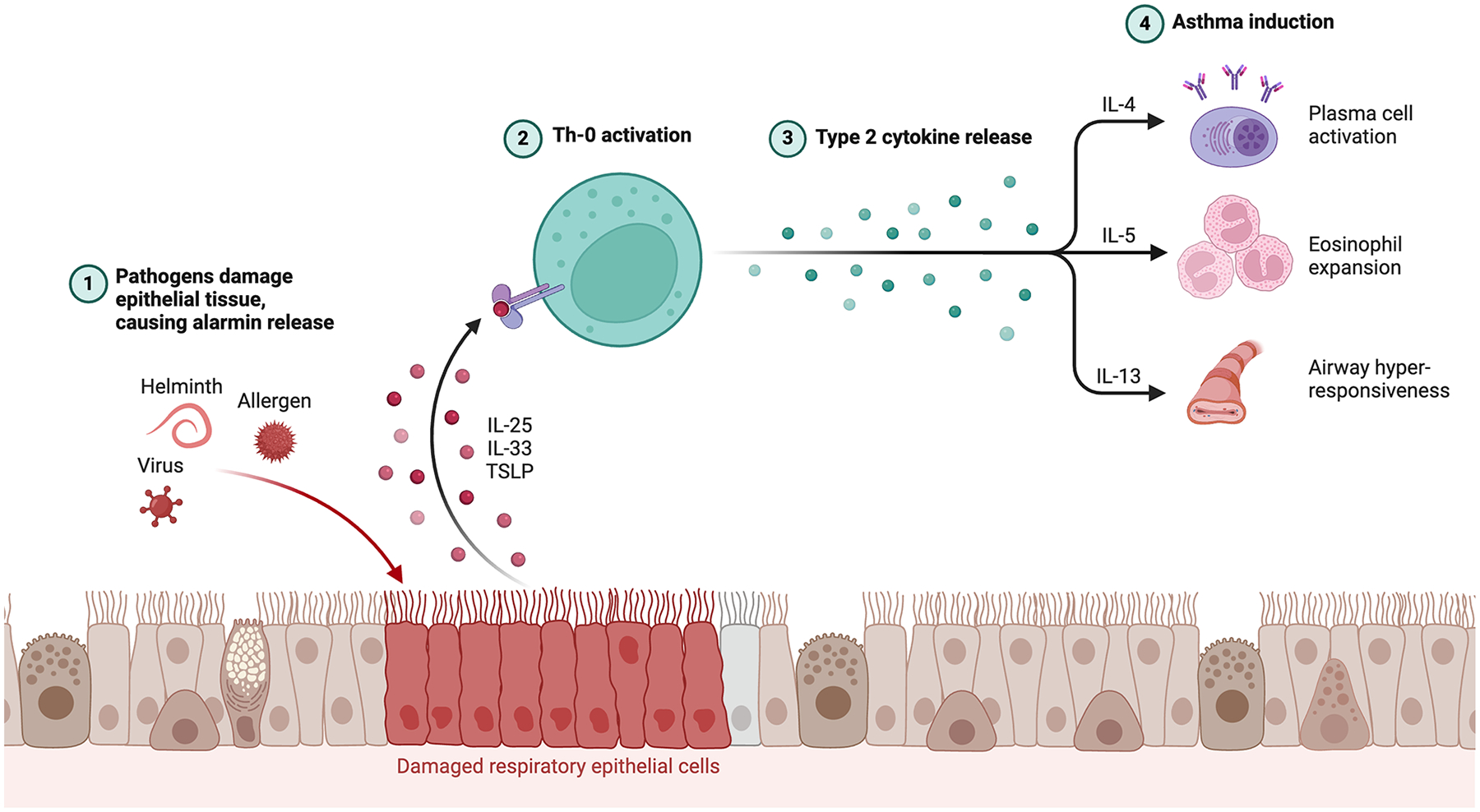
Pathogens such as viruses, helminths, or allergens damage the respiratory epithelium, leading to the release of epithelial-derived cytokines known as alarmins—IL-25, IL-33, and Thymic Stromal Lymphopoietin (TSLP). These alarmins activate naive CD4+ T cells (Th0 cells), promoting their differentiation into Th2 cells. Activated Th2 cells subsequently secrete type 2 cytokines. IL-4 promotes Th2 cell differentiation to induce IgE class switching necessary for allergic sensitization and mast cell activation while IL-13 is essential in mucus hypersecretion and airway hyperresponsiveness. On the other hand, IL-5 mainly recruits and activates eosinophils in the airways. Upon activation, eosinophils release toxic granules and inflammatory mediators that cause damage to the epithelium, contributing to airway remodeling that aggravates the severity of asthma, rendering the disease much more difficult to manage.

The pathology of asthma is based on the complex interaction between immune dysregulation, structural changes in the airways, and chronic inflammation (Figure 1). Important in this process is the activation of a series of immune cells, such as dendritic cells, eosinophils, mast cells, and T-helper 2 cells (Th2), which initiate an inflammatory cascade [6–14]. All these immune cells release a specific set of cytokines, predominantly IL-4, IL-5, and IL-13, playing vital roles in key features of asthma pathology [9, 10, 15–17].

Another hallmark of asthma pathology is structural changes in the airways, which further drive disease progression [18–23]. The destruction of the epithelium impairs its barrier function and increases susceptibility to environmental triggers such as allergens and pollutants. The excessive deposition of extracellular matrix leads to subepithelial fibrosis, which thickens the airway wall and reduces the lumen; thus, airway smooth muscle hypertrophy enhances bronchoconstriction and worsens airway stiffness [24]. Angiogenesis, the formation of new blood vessels, also contributes to vascular remodeling, which increases tissue edema and infiltration by inflammatory cells [25].

By the multifaceted release of histamine, leukotrienes, and prostaglandins, mast cells amplify inflammatory signaling, consequently aggravating the airway narrowing. Besides this, proteases such as tryptase may also take part in the processes of tissue remodeling and increased airway reactivity.

More recent studies in molecular biology have pointed out the involvement of non-coding RNAs in asthma pathology. For instance, long non-coding RNAs (lncRNAs), such as plasmacytoma variant translocation 1 (PVT1), have been described as critical modulators of airway smooth muscle (ASM) cell function [26]. The ASM cell is a particular cell type found in the airway linings of the lungs, primarily responsible for the functions of contraction and relaxation in the regulation of airflow through controlling the diameter of the air passages. Perry et al. [27] demonstrated that PVT1 regulates key ASM proliferation and contractility pathways, implicating its dysregulation in airway remodeling and disease severity.

### Genetic and Epigenetic Factors in the Pathogenesis of Bronchial Asthma

Asthma arises from a complicated interplay of genetic predispositions and environmental influences, producing a wide-ranging appearance of the disease varying enormously among individuals. Understanding these aspects is very critical for the clarification of the mechanisms participating in asthma and for developing appropriate personalized treatment interventions.

#### Genetic Factors:

Asthmatic susceptibility and exacerbation have already been reported as related to genetic predisposition. Gene variants encoding immune regulators, such as IL-4 and IL-13, had been linked to an increased chance of developing asthma; these cytokines orchestrate pathways of IgE class switching and Th2 differentiation, driving key pro-inflammatory features within the airway [28–33]. The research findings support that genetic variation in genes that regulate the immune response is associated with increased susceptibility to asthma and variable responses to treatment, especially in relation to corticosteroids [34].

Besides the immune-regulatory genes, genetic variations within glucocorticoid receptor signaling pathways are essential regulators of glucocorticoid therapies’ efficacies. Changes in the glucocorticoid receptor gene may obstruct the binding of the receptor to, and the succeeding anti-inflammatory signal, thus contributing to the worsening of GCI seen in severe asthma [35,36]. All these findings illustrate the crucial impact that genetic factors may impose on asthma phenotypes and treatment outcomes.

#### Environmental Factors:

Environmental exposures markedly increase asthma symptoms and impact the severity of the disease. Among the common allergenic triggers are dust mites, pollen, and animal dander, which initiate and perpetuate inflammatory responses [2, 3, 37]. Respiratory viral infections, especially rhinoviruses, are significant precipitants of asthma exacerbations, involving the activation of dendritic cells, eosinophils and other immune cells, involving toll-like receptors and other cell surface molecules causing acute inflammation and airway obstruction [38–43].

Air pollution is one of the major environmental factors that exacerbate asthma through both short-term and long-term mechanisms. Pollutants like particulate matter (PM2.5) and nitrogen dioxide (NO₂) induce oxidative stress in the epithelial cells of the airways, which enhances the production of pro-inflammatory cytokines and chemokines [44]. Kelly et al. [45] demonstrated that exposure to PM2.5 disrupts glucocorticoid receptor signaling pathways and impairs glucocorticoid function in the exacerbation of airway inflammation in predisposed individuals. These pollutants increase the symptoms of asthma and cause long-term airway remodeling and progression of the disease.

The complex etiology of asthma is further compounded by the synergistic relationship between genetic and environmental factors. For example, individuals with genetic predispositions that result in increased immune responses are more likely to develop severe asthma when exposed to environmental stimuli such as pollutants or allergens. Their interaction underlines the need to understand both the molecular and environmental determinants of asthma to guide effective prevention and treatment approaches [46].

### Current Treatment Strategies in Bronchial Asthma

Asthma requires an individualized strategy of management with a view to decreasing inflammation, symptom relief, and prevention of exacerbation. Generally, the management follows a stepwise approach: increasing treatment intensity with the rising degree of disease severity.

Short-acting beta-agonists (SABAs) are the first-line treatment for the rapid relief of bronchoconstriction during acute asthma exacerbations. These agents act on β2-adrenergic receptors in airway smooth muscle, inducing relaxation and improving airflow. Although effective in the immediate control of symptoms, SABAs do not treat the underlying inflammation and hence are not appropriate for long-term management [47]. Moreover, the (s)-isomer of SABAs may induce pro-constrictory and pro-inflammatory pathways in the airway smooth muscle cells [18].

For this reason, effective symptom control usually is achieved by adding long-acting beta-agonists (LABAs) to corticosteroids [48]. LABAs provide sustained bronchodilation that can relieve nocturnal symptoms and improve overall pulmonary function. However, LABAs are not for use as monotherapy because they may lead to worsening of inflammation without the possibility of its control at the root cause [49]. Adjunct therapies, such as vitamin D supplementation to enhance immunity, have also been suggested [50–52].

Inhaled corticosteroids (ICS) are the cornerstone of asthma management. These drugs work through an effect on glucocorticoid receptor signaling pathways, reducing airway inflammation. ICS reduces the production of pro-inflammatory cytokines, inhibits inflammatory cell recruitment, and restores the integrity of the airway epithelium [53]. The use is most effective in patients with mild to moderate asthma, resulting in improvements in both symptom control and lung function. However, add-on therapies are often necessary in patients with severe asthma or GCI, since these patients have lost responsiveness to ICS. Systemic or oral corticosteroids are generally reserved for severe asthma or acute exacerbations unresponsive to inhalation treatments. Systemic corticosteroids are effective in the control of severe inflammation but have a high risk of side effects, such as adrenal suppression, osteoporosis, hyperglycemia, and metabolic dysregulation, which precludes their long-term use [54].

Leukotriene receptor antagonists (LTRAs) target leukotriene-mediated inflammation, which is more prominent in exercise-induced asthma and aspirin-exacerbated respiratory disease [55]. These agents reduce bronchoconstriction, mucus secretion, and inflammation by blocking leukotriene receptors in airway smooth muscle and immune cells.

Biologic therapies represent a major advance in the treatment of severe asthma subtypes with specific inflammatory mechanisms. The administration of targeted biologic agents such as mepolizumab and benralizumab block IL-5 signaling—an important determinant of eosinophil recruitment and survival, and thereby decrease eosinophilic inflammation [56]. Dupilumab is an anti-IL-4/IL-13 drug acting on Th2-mediated inflammation and leading to reduced mucus production and airway remodeling.

These treatment modalities are particularly useful in patients with glucocorticoid-resistant asthma, offering a steroid-sparing option for long-term management [57]. The evolution of asthma management underlines the requirement for precision medicine approaches, which address the heterogeneous features of the disease. Innovations in biologics and combination therapies keep on transforming asthma management and continue to bring fresh hope to patients with severe or refractory asthma.

### Glucocorticoids and their Receptor-induced Cellular Signaling

Glucocorticoids (GCs) belong to a class of steroid hormones commonly used in the treatment of inflammatory and immune-mediated diseases, such as asthma [58]. Characterized by their prominent anti-inflammatory property, GCs have been considered a cornerstone in the treatment of asthma. The molecular basis for their mode of action is the modulation of gene transcription; they not only inhibit pro-inflammatory mediators but also activate the transcription of anti-inflammatory elements. Together, these effects make GCs very potent in the suppression of airway inflammation and hyperresponsiveness and prevention of exacerbation in asthmatic cases.

Clinically, GCs are given either as inhaled corticosteroids or systemic corticosteroids, depending on the severity of the disease. ICS, such as budesonide and fluticasone, have local anti-inflammatory actions while reducing systemic adverse effects; thus, these drugs become the first choice for many patients with asthma. Systemic corticosteroids, such as prednisone, are reserved for use in the very severe or acute exacerbation cases because of the generalized immunosuppressive action and the possibility of systemic complications associated with them.

### Mechanisms of Glucocorticoid Action

**Figure 2:**
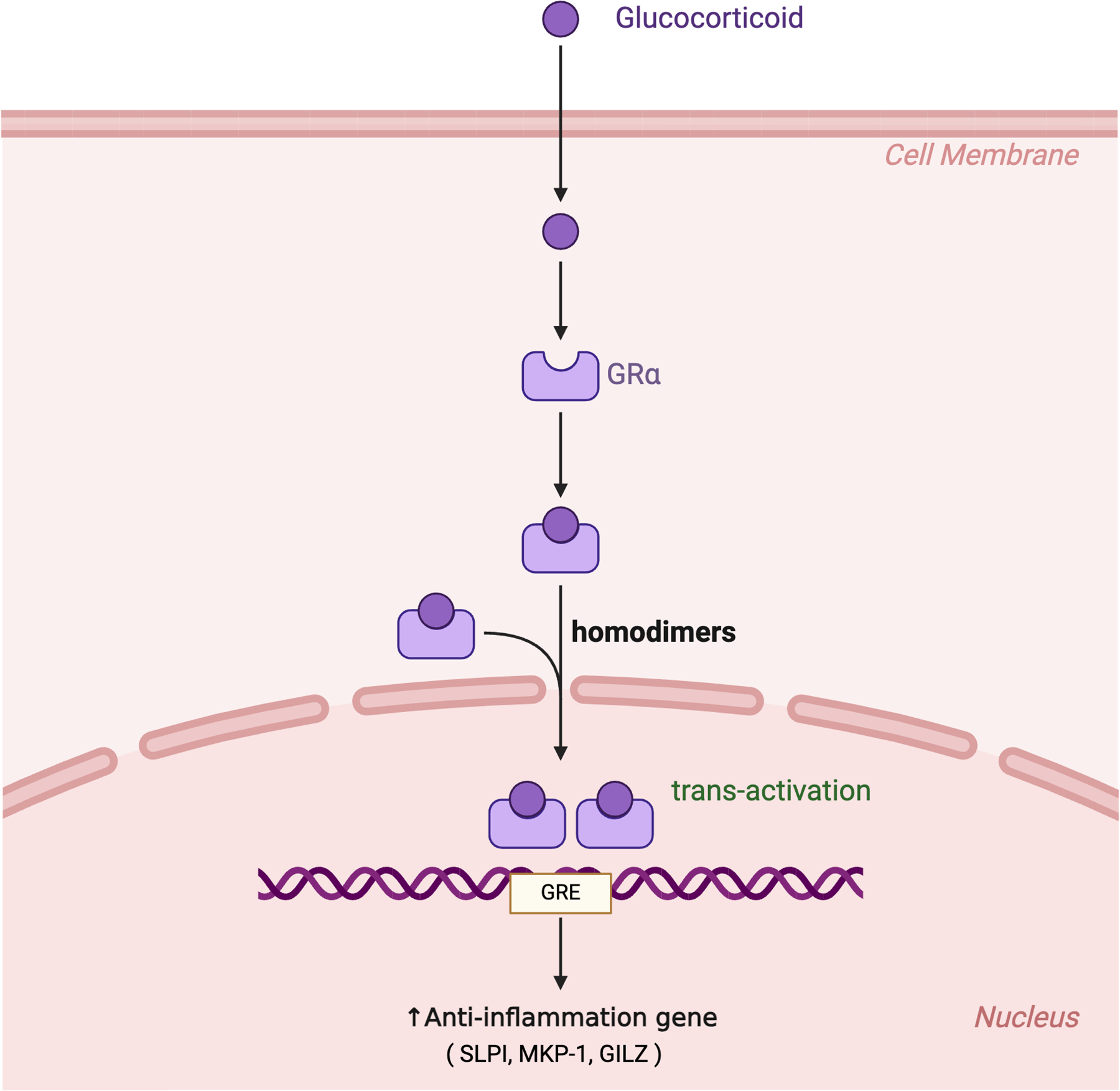
Glucocorticoids enter the cell and bind to the glucocorticoid receptor alpha (GRα) in the cytoplasm. The resulting complex then moves into the nucleus, where GRα forms homodimers that bind to glucocorticoid response elements (GREs) within glucocorticoid-responsive genes. This binding can lead to the transcriptional activation of genes that produce anti-inflammatory proteins, including secretory leukocyte protease inhibitor (SLPI), mitogen-activated protein kinase phosphatase-1 (MKP-1), and glucocorticoid-induced leucine zipper (GILZ).

Glucocorticoids exert their therapeutic effect in asthma through binding to the glucocorticoid receptor (GR), a ligand-activated transcription factor expressed by all airway cells. These interactions induce two interdependent yet distinct molecular mechanisms: GR transactivation and GR transrepression, both of which are involved in controlling the inflammation and immune response characteristically seen in asthma (Figure 2).

### GR Transactivation

Transactivation refers to the direct stimulation of transcription for genes with anti-inflammatory effects. Glucocorticoids move through the lipid bilayer and bind to cytoplasmic glucocorticoid receptors [59]. This binding induces a conformational change in the receptor that triggers its dissociation from bound chaperone proteins, such as heat shock protein 90 (HSP90) [60]. The activated glucocorticoid-glucocorticoid receptor (GC-GRα) complex then translocates into the nucleus where it binds to specific DNA sequences known as glucocorticoid response elements (GREs) [59]. This binding can lead to the transcriptional activation of genes that produce anti-inflammatory proteins, including secretory leukocyte protease inhibitor (SLPI), mitogen-activated protein kinase phosphatase-1 (MKP-1), and glucocorticoid-induced leucine zipper (GILZ) (Figure 2).

It also upregulates the transcription of anti-inflammatory genes, including IL-10 and annexin-1, which are involved in the inhibition of pro-inflammatory signaling cascades. IL-10 inhibits the release of pro-inflammatory cytokines from immune cells [61], while annexin-1 modulates neutrophil recruitment and vascular permeability [62]. Through these actions, transactivation mechanisms restore inflammatory balance and promote airway homeostasis.

### GR Transrepression

Contrary to transactivation, GR transrepression is the repression of pro-inflammatory gene expression through interference with important transcription factors. GC-GR complexes inhibit the activities of nuclear factor-κB (NF-κB) and activator protein-1 (AP-1) [63], without which the expression of cytokines and chemokines is vital. The mechanism is protein–protein interaction rather than direct DNA binding; therefore, genes that produce inflammatory mediators, such as IL-4, IL-5, and TNF-α, are prevented from transcription.

It is through transrepression that GCs reduce airway inflammation, targeting cytokine and chemokine production, leukocyte recruitment, and expression of vascular adhesion molecules. This mechanism complements the effects of transactivation, thus creating a wide-ranging anti-inflammatory response.

### Effects of Glucocorticoids on Airway Cells

Glucocorticoids have profound effects on airway epithelial cells, which are pivotal in the pathophysiology of asthma. They reduce inflammation by inhibiting the synthesis of pro-inflammatory cytokines and chemokines, in large part through inhibition of the NF-κB signaling pathway. GCs enhance the integrity of the epithelial barrier by increasing the expression of tight junction proteins, facilitating the repair of injured epithelium [64], and decreasing permeability to allergens. They also reduce airway remodeling by inhibiting epithelial-mesenchymal transition and suppressing the production of growth factors such as epidermal growth factor (EGF) and vascular endothelial growth factor (VEGF) [65]. GCs also reduce mucus hypersecretion by decreasing goblet cell hyperplasia and downregulating MUC5AC expression [66]. They lower oxidative stress in AECs by enhancing antioxidant defenses and reducing reactive oxygen species (ROS) production. Moreover, GCs promote anti-inflammatory mediators, such as annexin A1, and inhibit epithelial-driven processes that contribute to chronic inflammation and remodeling. However, limitations such as GC insensitivity, particularly in severe asthma or exacerbations, and potential impairment of antiviral responses underline the complexity of their effects on AECs in asthma management [67].

## Glucocorticoid Insensitivity (GCI)

### Clinical Definition of GC Insensitivity

Glucocorticoid insensitivity is pragmatically defined as a failure of the forced expiratory volume in one second (FEV1) to increase by more than 10–15% following corticosteroid therapy, with its prevalence highest in patients with severe asthma and this subpopulation expressing the feature most often, between 5% and 10% [59]. It appears to be linked to poor control of symptoms and persistent inflammation of the airways, consequently increasing morbidity. Other than asthma, GCI has also been reported in several other diseases characterized by inflammation, such as COPD and rheumatoid arthritis, thus showing its wide clinical significance among diseases [68].

### Primary vs. Acquired GCI

GCI can be categorized into two distinct subtypes: primary (intrinsic) and acquired, each driven by different mechanisms, as discussed below.

### Primary (Intrinsic) GCI

**Figure 3:**
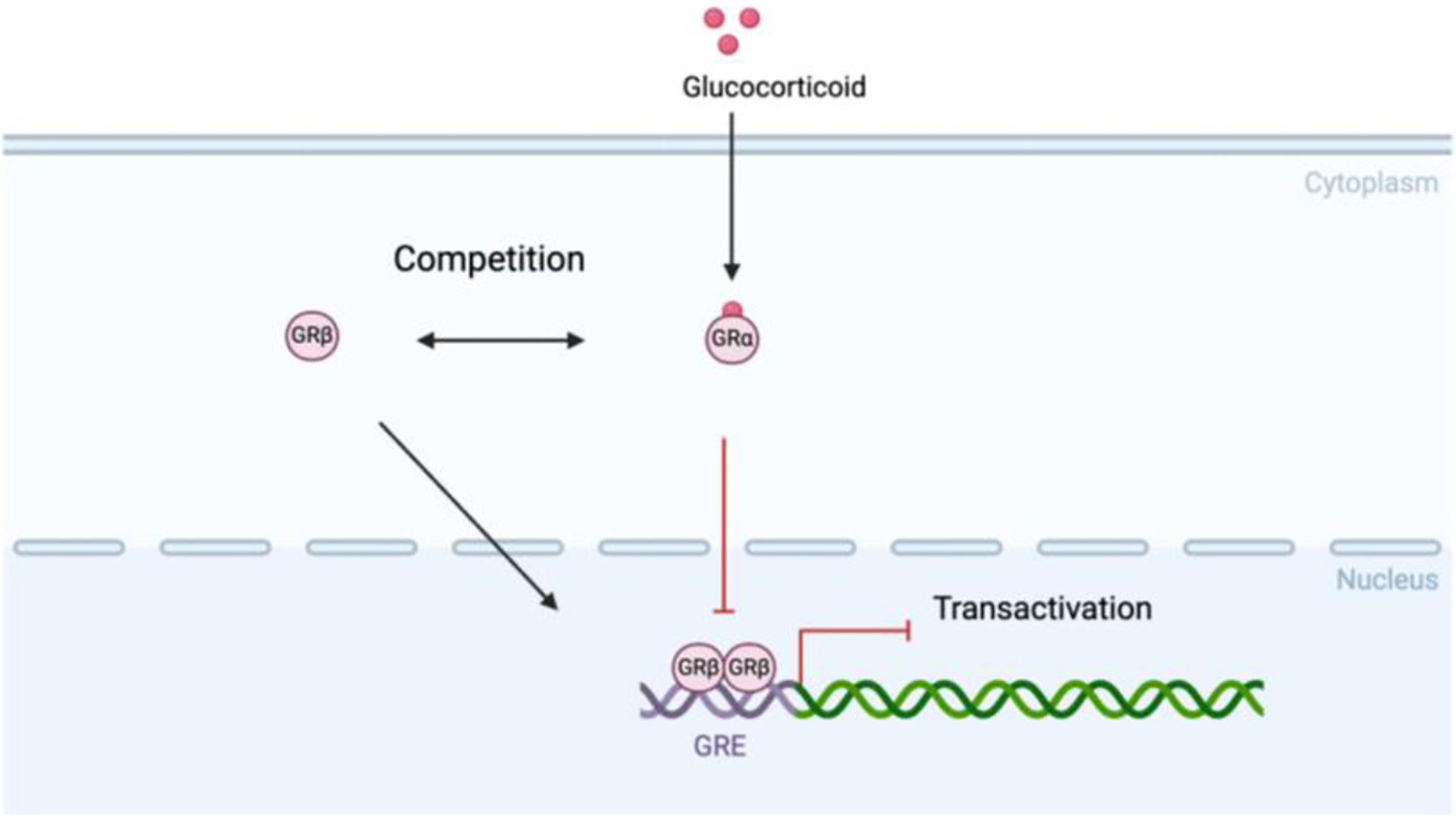
GR-β, which does not bind to glucocorticoids, can still translocate to the nucleus and bind to GREs. GR-β competes with GR-α for GRE binding sites, interfering with GR-α-mediated gene transactivation. This competitive inhibition impairs the genomic actions of glucocorticoids, leading to reduced therapeutic efficacy and the development of glucocorticoid resistance.

Primary glucocorticoid insensitivity is a lifelong and intrinsic resistance to glucocorticoid therapy, often genetically determined. One of the important molecular mechanisms contributing to primary GCI is the overexpression of the glucocorticoid receptor β (GRβ) isoform [69]. The GRβ isoform is a dominant-negative receptor that competes with the active GRα isoform, thus markedly inhibiting the transcription of genes involved in anti-inflammatory responses [59] (Figure 3). Moreover, genetic changes or post-translational modifications of the glucocorticoid receptor itself—such as altered phosphorylation—impair its ability to bind to DNA and promote anti-inflammatory actions. Dysregulation of GR signaling pathways, including changes in GR phosphorylation, makes a substantial contribution to the inherent glucocorticoid resistance in severe asthma [26].

### Acquired GCI

Acquired GCI develops over time due to external factors that interfere with glucocorticoid efficacy. Chronic smoking is a major contributor, as tobacco smoke causes oxidative stress and activates inflammatory pathways such as NF-κB, which oppose GR-mediated anti-inflammatory actions. Respiratory viral infections, especially rhinovirus, are well known to exacerbate airway inflammation and further compromise glucocorticoid responsiveness. Environmental pollutants such as particulate matter (PM2.5) impair GR signaling by inducing systemic inflammation and oxidative stress. These findings emphasize the multicausal nature of acquired GCI, its reliance on environmental and behavioral risk factors from outside [45].

### Involvement of Immune vs. Non-Immune Cells

GCI involves dysregulated GR signaling in both immune and non-immune (structural) airway cells, which perpetuates inflammation and airway remodeling.

### Immune Cell Involvement

In immune cells, such as macrophages, T-cells, and dendritic cells, overexpression of GRβ plays a critical role in suppressing the anti-inflammatory effects of GRα. Additionally, pro-inflammatory transcription factors, including NF-κB, activator protein-1 (AP-1), and interferon regulatory factor-1 (IRF-1), competitively interact with GR for binding sites on DNA as well as for transcriptional coactivators. Such competitive interaction limits GR’s ability to suppress the production of pro-inflammatory cytokines, such as IL-4, IL-5, and TNF-α. This interference negatively affects GR-mediated transactivation, ultimately decreasing the effectiveness of glucocorticoids [70]. Increased kinase activity, in particular p38 MAPK and JNK, further phosphorylates GR at inhibitory sites, which decreases its nuclear translocation and DNA-binding capacity. Alterations in phosphatase activity, such as a decrease in the function of protein phosphatases like PP2A and PP5, augment these effects and contribute to GCI [69].

### Non-Immune (Structural) Cell Involvement

In nonimmune cells, such as airway smooth muscle and airway epithelial cells, GCI disrupts key control mechanisms. Inside the epithelium, these GCs impact GR-mediated pathways that result in failure to switch off proinflammatory cytokines such as IL-33 and chemokines including eotaxin. In this way, mediators responsible for the engagement of immune cells facilitate inflammation within an already inflamed airway. ASM cells, which are directly involved in airway remodeling, show a diminished response to GC-induced suppression of pro-inflammatory gene expression. In severe asthma, there is a dysregulation of the NF-κB and MAPK pathways, whereby the ASM cells can proliferate and contribute to airway remodeling despite corticosteroid treatment [71].

### Other Mechanisms of Glucocorticoid Insensitivity

The mechanisms contributing to GCI are not restricted to localized airway inflammation but also include systemic, genetic, epigenetic, and metabolic factors that interfere with glucocorticoid receptor signaling and reduce the effectiveness of glucocorticoid treatments. Such factors increase airway inflammation and add to the challenges in managing severe asthma.

### HPA Axis Dysfunction

**Figure 4:**
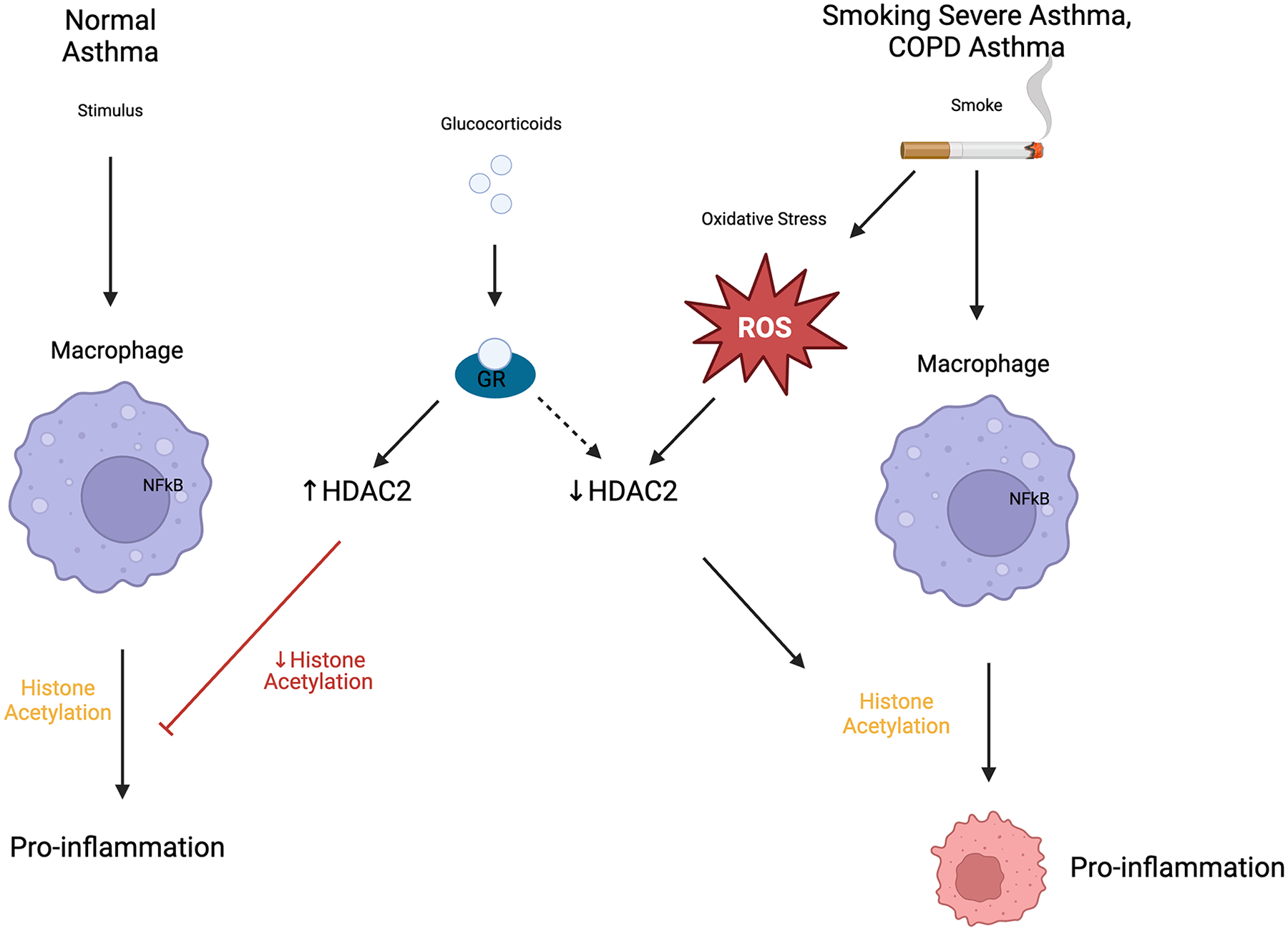
In a normal asthma patient, glucocorticoids recruit HDAC2 to the NF-κB inflammatory gene complex, which helps to silence these genes, thereby reducing inflammation. Oxidative stress, a consequence of cigarette smoke or severe asthma, can impair HDAC2 activity, amplifying the inflammatory response to NF-κB activation and reducing the anti-inflammatory effects of corticosteroids.

The hypothalamic-pituitary-adrenal (HPA) axis is central to the regulation of systemic glucocorticoid levels, and its dysregulation is a major contributor to GCI. Chronic stress disturbs cortisol production and receptor regulation, leading to the downregulation of GR expression and function [72]. Prolonged systemic glucocorticoid administration also suppresses adrenal function [73], lowers endogenous cortisol levels, and changes the dynamics of GR signaling. This repression is further enhanced by downregulation of histone deacetylase 2 (HDAC2), an enzyme critical for glucocorticoid receptor-mediated transcriptional activity (Figure 4). Further studies showed that the recovery of HDAC2 function could open possibilities for therapeutic intervention in reversing these effects [74].

### Epigenetic Modifications

Epigenetic changes, specifically DNA methylation and histone acetylation, have recently been shown to be important modulators of GR expression and activity. High methylation levels of GR promoter regions reduce the expression of GR, which impairs glucocorticoid responsiveness. Histone acetylation patterns also significantly influence chromatin accessibility for GR-DNA binding. Changes in these patterns disrupt the function of GR in activating anti-inflammatory genes. Restoration of HDAC2 activity has been associated with reversal of epigenetic changes, which restored glucocorticoid sensitivity [75].

### Metabolic Dysregulation

Obesity and related metabolic derangements are strong contributors to systemic glucocorticoid resistance. In obesity, fat tissue secretes various proinflammatory substances, such as adipokines – leptin and resistin – and cytokines – IL-6 that collectively induce a status of chronic low-grade inflammation in the body. Such an inflammatory milieu interferes with glucocorticoid receptor signaling and impairs the glucocorticoid anti-inflammatory effect [76].

Adipose tissue inflammation, characterized by rises in ROS and free fatty acid, further aggravates GR phosphorylation and nuclear translocation, worsening glucocorticoid resistance [77]. These data demonstrate the interconnectedness of metabolic and glucocorticoid efficacy and that a holistic approach to asthma treatment is necessary.

### Drug Interactions

Some drugs also affect glucocorticoid responsiveness by direct or indirect pathways. Chronic exposure to β2-adrenergic agonists desensitizes GR signaling by modifying the interaction between β-adrenergic and glucocorticoid pathways [78]. This desensitization reduces the efficacy of combination therapy using glucocorticoids plus β2-agonists. Systemic antibacterial drugs, by changing the composition of gut microbiota, may indirectly modulate systemic inflammation and immunological function [79] and, therefore, affect GR functions. These interactions illustrate the need for careful pharmacological management in patients with severe asthma.

## Clinical Implications of Glucocorticoid Insensitivity

### Challenges in GCI Asthma

Glucocorticoid insensitivity is one of the important problems in the treatment of severe asthma. Since conventional therapies—both inhaled and systemic corticosteroids—do not control airway inflammation well enough in patients with glucocorticoid insensitivity, the latter is going to persist with symptoms, frequent exacerbations, and increased health care utilization. Failure of glucocorticoids to suppress pro-inflammatory pathways, including NF-κB and STAT3, enhances airway remodeling, mucus hypersecretion, and hyperresponsiveness [67,71].

Those with GCI asthma often have comorbidities, such as obesity, systemic inflammation, and chronic infections, which can further contribute to glucocorticoid resistance and make management difficult. The long-term administration of high doses of systemic corticosteroids is associated with increased risk of side effects such as adrenal suppression, osteoporosis, and metabolic dysregulation [80]; hence, there has been an unmet need for alternative therapeutic strategies.

### Novel Treatments for GCI Asthma

Advances in molecular and pharmacological research have now yielded targeted therapies to overcome glucocorticoid resistance by either restoring GR function or bypassing disrupted pathways to achieve effective disease control.

### JAK Inhibitors

**Figure 5:**
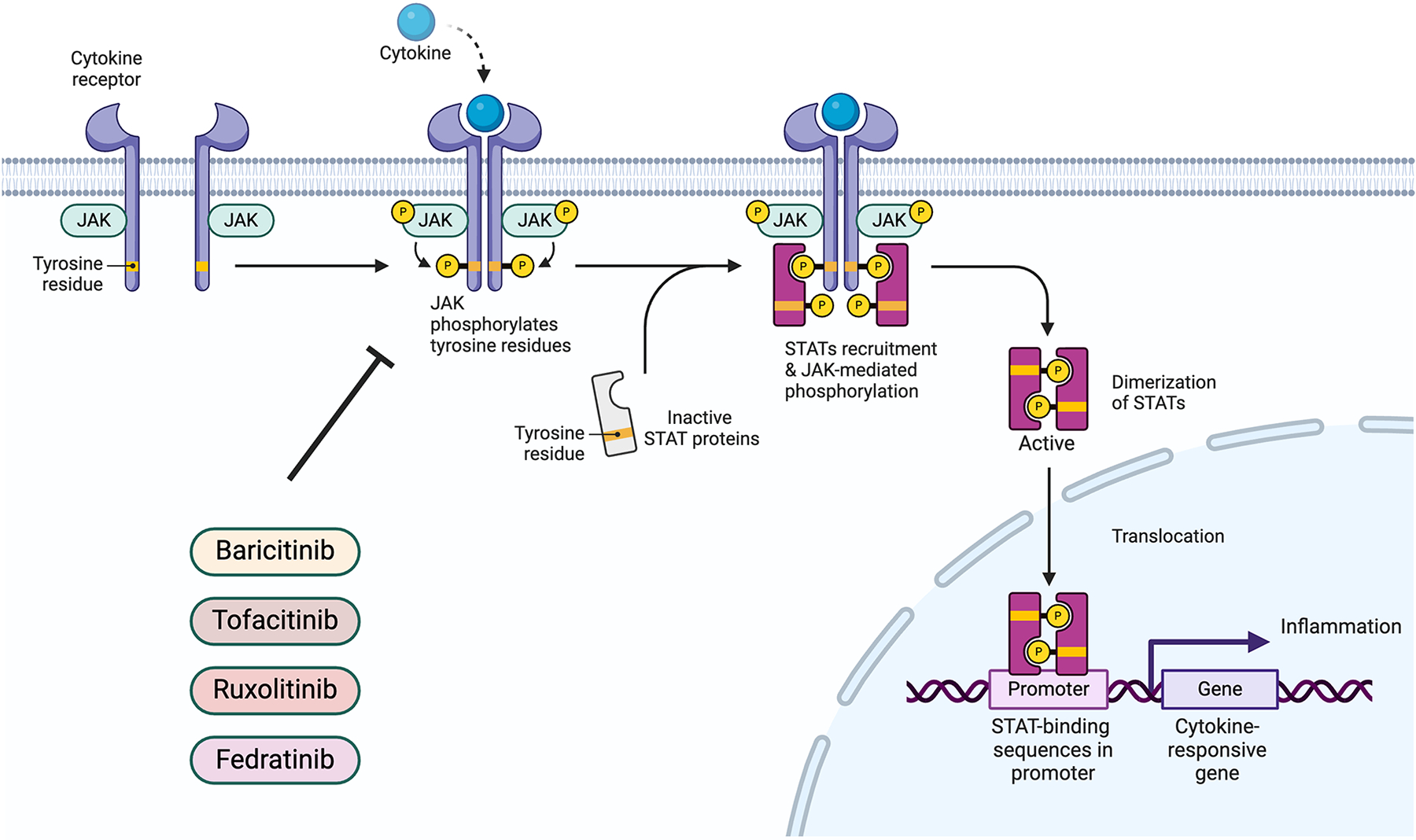
The human JAK family, together with the STATs, are major contributors to the transmission of signals from extracellular receptors to the cell nucleus. Phosphorylated STATs regulate interferon-induced gene expressions, lymphocyte differentiation and proliferation, and participate in cytokine-induced immune overreaction, leading to, ultimately, inflammatory tissue injury at an organ level that may also act at the systemic level.

Janus kinase (JAK) inhibitors, such as tofacitinib, are a new class of drugs under development for cytokine-driven inflammation mediated by IL-6/STAT3 signaling (Figure 5). This pathway has been implicated in glucocorticoid resistance in asthma, particularly in neutrophilic phenotypes of the disease characterized by increased numbers of neutrophils in the sputum. Blockade of JAK activity reduces pro-inflammatory cytokine production and diminishes both systemic and airway inflammation, restoring glucocorticoid responsiveness. Clinical studies have demonstrated that JAK inhibitors reduce exacerbations and improve lung function significantly in patients with cytokine-driven GCI asthma and COVID-19-induced exacerbations [71, 81, 82]. These data highlight their potential as a promising therapeutic approach in severe, treatment-resistant asthma (Figure 5).

### Biologics

Biologic therapies have revolutionized the treatment of severe asthma by providing precision-targeted interventions on specific inflammatory pathways. The target of anti-IL-5 therapies, such as mepolizumab and benralizumab, is eosinophilic inflammation via IL-5 inhibition—a cytokine critical to eosinophil recruitment and survival [83]. These biologics have been shown to reduce exacerbations and improve lung function in patients with eosinophilic asthma [84]. Anti-IL-4/IL-13 therapies, including dupilumab, block Th2-driven inflammation by targeting IL-4 and IL-13 signaling pathways [85]. Such therapies may also alleviate airway hyperresponsiveness and remodeling, offering symptom relief in severe asthma phenotypes. Anti-TNF-α therapies target systemic inflammation and have been promising in restoring glucocorticoid sensitivity, especially in obesity-related GCI asthma. These biologics, acting as anti-TNF-α, offer a reduction in systemic inflammatory load and improvement in asthma outcomes [86].

### Small-Molecule Drugs

Small-molecule drugs targeting disrupted GR pathways offer another therapeutic avenue in GCI asthma.

HDAC2 activators increase the activity of histone deacetylase 2, an essential mediator of GR function. Restoring HDAC2 levels, these drugs reverse glucocorticoid resistance induced by oxidative stress and inflammation. Similarly, agents targeting DNA methylation patterns are under investigation for their potential to modulate the epigenetic landscape and address the molecular drivers of GCI. The results of this approach in preclinical and early-phase clinical studies have been encouraging [85].

**Figure 6:**
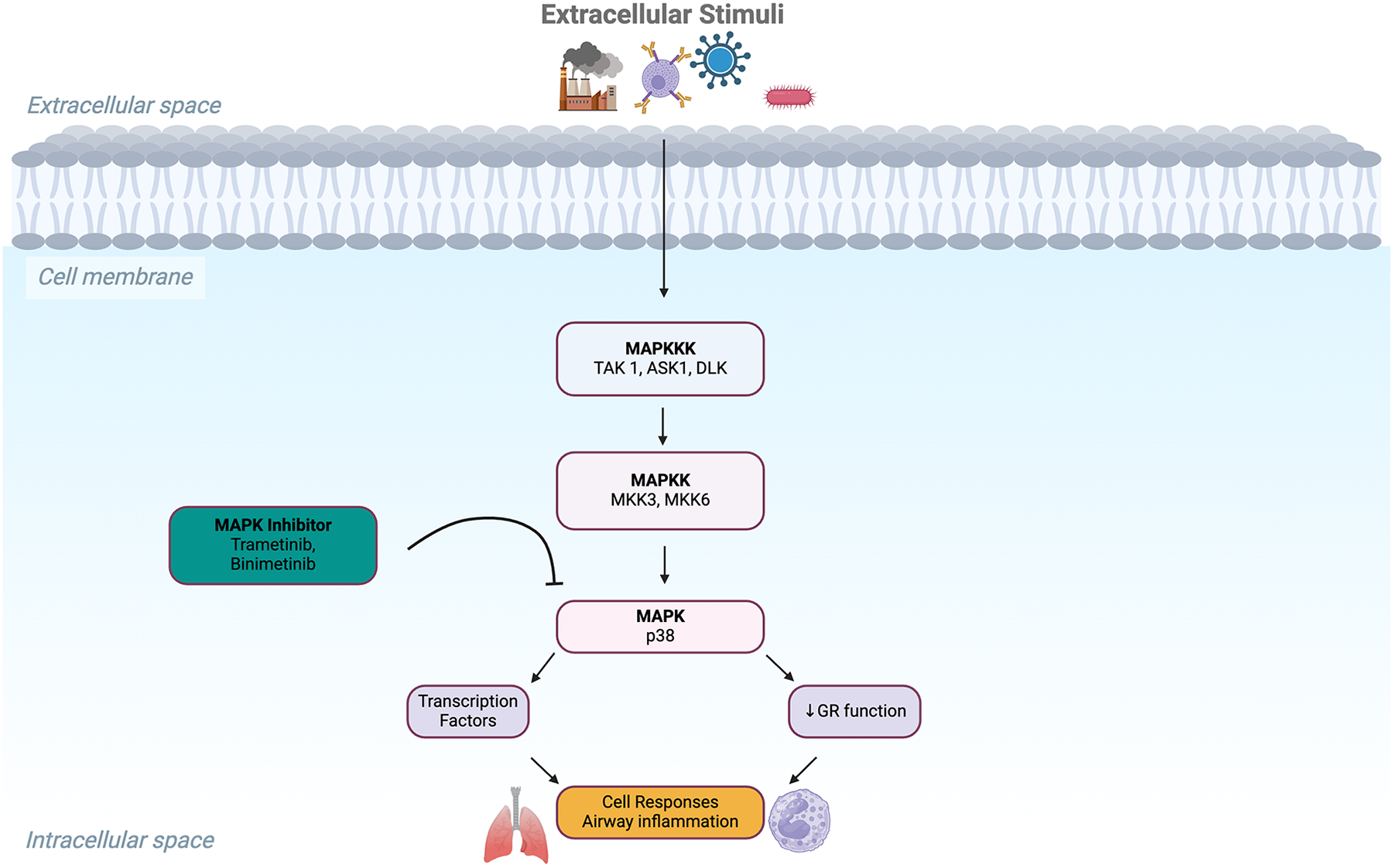
Stimuli trigger a cascade of phosphorylation events beginning with MAP kinase kinase kinases (MAPKKK), including TAK1, ASK1, and DLK. These activate MAP kinase kinases (MAPKK), specifically MKK3 and MKK6, which phosphorylate and activate p38 MAPK. Activated p38 MAPK translocates to the nucleus, modulating transcription factors that contribute to pro-inflammatory gene expression. This leads to increased airway inflammation and reduced glucocorticoid receptor function, contributing to corticosteroid insensitivity in asthma. MAPK inhibitors such as Trametinib and Binimetinib target upstream kinases (MAPK), aiming to block this signaling cascade. Inhibiting this pathway may help restore corticosteroid sensitivity and reduce airway inflammation in severe asthma.

MAPK Inhibitors target p38 MAPK, a kinase that, when activated, phosphorylates GR at inhibitory sites, impairing its nuclear translocation and transcriptional activity (Figure 6). By inhibiting p38 MAPK activity, these compounds restore GR function, with an enhancement in the anti-inflammatory effects of glucocorticoids [67, 87]. SYK Inhibitors block spleen tyrosine kinase (SYK), reducing mast cell-mediated inflammation and improving asthma control. This mechanism offers a novel approach for patients with steroid-resistant asthma [88].

### IL-10 Replacement Therapy

Interleukin-10 (IL-10), an anti-inflammatory cytokine with a strong effect, takes part in modulating immune reactions and maintaining homeostasis of the airways. Targeting IL-10 has been shown to reduce pro-inflammatory cytokine production and could restore glucocorticoid receptor sensitivity in some animal models of steroid-resistant asthma. This approach may be an effective adjunct therapy for the control of inflammation in glucocorticoid-insensitive asthma [74].

## Conclusion

Glucocorticoid insensitivity is one of the significant barriers to effective treatment of severe asthma, and it limits therapeutic response to conventional glucocorticoid-based therapy, resulting in poor disease outcomes. The multifactorial mechanisms contributing to GCI include perturbations in glucocorticoid receptor signaling, increased activation of pro-inflammatory cytokine pathways, systemic inflammation, and epigenetic changes. These perturbations cause persistent airway inflammation, structural remodeling, and impaired corticosteroid responsiveness, thus posing great challenges in disease management.

The future of asthma management is increasingly taking a precision approach, accounting for the heterogeneity in GCI and its underlying molecular mechanisms. Accordingly, the identification of reliable biomarkers, such as GRβ expression and STAT3 activation, cytokine profiles, among others, that can enable an early diagnosis and guide personalized management of GCI holds great promise, and these biomarkers may also evaluate therapeutic responses relevant to improving outcome in patients with asthma.

The progress noted in biologic treatments, mainly the increased usage of dupilumab and mepolizumab, will likely continue to shift the therapeutic landscape for severe asthma. Further therapeutic strategies aiming at JAK/STAT and MAPK signaling pathways and epigenetics hold promise in targeting the molecular roots of GCI. Further studies on systemic inflammatory processes and metabolic dysregulation—especially related to obesity-associated asthma—could help identify further targets and novel approaches to achieve better disease control.

In conclusion, such integration into the individualized therapeutic models may offer hope for changing the face of GCI management, reducing the burden of disease and improving the quality of life for patients with severe, treatment-resistant asthma. Addressing the molecular, genetic, and environmental determinants of GCI, health care can move toward more effective, patient-centered care, easing the global burden of severe asthma.

## Key Points

- Definition and Clinical Significance of GCI: Glucocorticoid insensitivity (GCI) refers to a <10–15% improvement in FEV1 despite adequate glucocorticoid therapy, particularly affecting 5–10% of severe asthma patients and contributing to poor disease control.
- Molecular Basis of GCI: GCI arises from impaired glucocorticoid receptor (GR) signaling, including overexpression of GRβ (a dominant-negative isoform), increased activity of NF-κB and AP-1, and abnormal GR phosphorylation via kinases like p38 MAPK.
- Primary vs. Acquired GCI: Primary (intrinsic) GCI is genetically driven (e.g., GR mutations or GRβ overexpression), whereas acquired GCI results from environmental stressors like smoking, pollution, viral infections, or chronic stress altering GR signaling.
- Cellular Involvement in GCI: Immune cells (macrophages, T cells) and non-immune cells (epithelial and smooth muscle cells) contribute. These cells show impaired response to GCs due to disrupted GR function and heightened inflammatory signaling.
- Role of Epigenetics and Metabolism: Epigenetic modifications (e.g., DNA methylation, histone acetylation) and metabolic disturbances (e.g., obesity-related inflammation) significantly impair GR-mediated anti-inflammatory responses.
- HPA Axis and Systemic Factors: HPA axis dysfunction from chronic stress or long-term steroid use downregulates GR and HDAC2 expression, worsening glucocorticoid response and airway inflammation.
- Current Treatment Limitations: Standard therapies like ICS and systemic steroids are ineffective in GCI, leading to persistent inflammation, remodeling, and frequent exacerbations with adverse side effects from high-dose steroids.
- Biologic Therapies: Biologics targeting IL-5 (mepolizumab, benralizumab), IL-4/IL-13 (dupilumab), and TNF-α offer personalized options for patients with eosinophilic and Th2-driven asthma phenotypes resistant to steroids.
- Emerging Therapies for GCI: JAK inhibitors (e.g., tofacitinib), MAPK inhibitors, HDAC2 activators, and SYK inhibitors show effective treatment in restoring steroid responsiveness by targeting inflammatory or epigenetic pathways.
- Future Directions: There is a need for further careful studies in the identification of the reliable biomarkers, molecular, genetic, and environmental determinants, and systemic inflammatory processes and metabolic dysregulation of GCI.
